# Supplementary material for: Investigation of the Protective Effect for GcMAF by a Glycosidase Inhibitor and the Glycan Structure of Gc Protein
Source: Molecules. 2023 Feb 6;28(4):1570. doi: 10.3390/molecules28041570 (PMC9963009; doi:10.3390/molecules28041570)
Supplement: Supplementary file 1 [file molecules-28-01570-s001.zip › molecules-2139593-supplementary.pdf]

molecules

Supplementary material

Investigation of the Protective Effect for GcMAF by a Glycosidase Inhibitor and the Glsycan Structure of Gc Protein

Yoshimi Kanie [1], Yuya Maegawa [2], Yi Wei [3] and Osamu Kanie [2,3,4,*]

[1] Research Promotion Division, Tokai University, 4-1-1 Kitakaname, Hiratsuka, Kanagawa, 259-1292, Japan
[2] Department of Applied Biochemistry, Tokai University, 4-1-1 Kitakaname, Hiratsuka, Kanagawa 259-1292 Japan
[3] Graduate School of Science and Technology, Tokai University, 4-1-1 Kitakaname, Hiratsuka, Kanagawa 259-1292 Japan
[4] Micro/Nano Technology Center, Tokai University, 4-1-1 Kitakaname, Hiratsuka, Kanagawa 259-1292 Japan; Graduate School of Science and Technology, Tokai University, 4-1-1 Kitakaname, Hiratsuka, Kanagawa 259-1292 Japan
* Correspondence: kanie@tokai-u.jp (O.K.); Tel.: +81 463 63 4613 (O.K.)

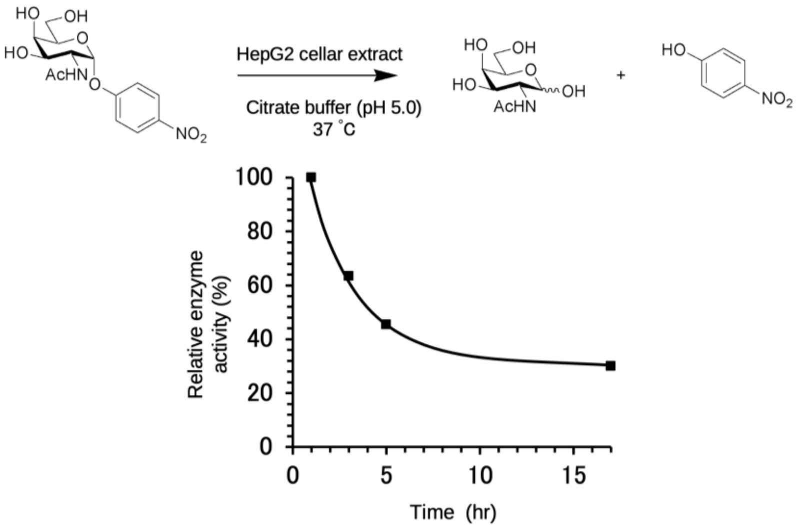

Figure. S1. Time course study of α-GalNAc-ase activity of HepG2 cellular extract.
1 mM PNP-α-GalNAc and the cellular extract were incubated at 37 °C in 50 mM citrate buffer (pH 5.0). The half of α-GalNAc-ase activity diminished within 4 hours.

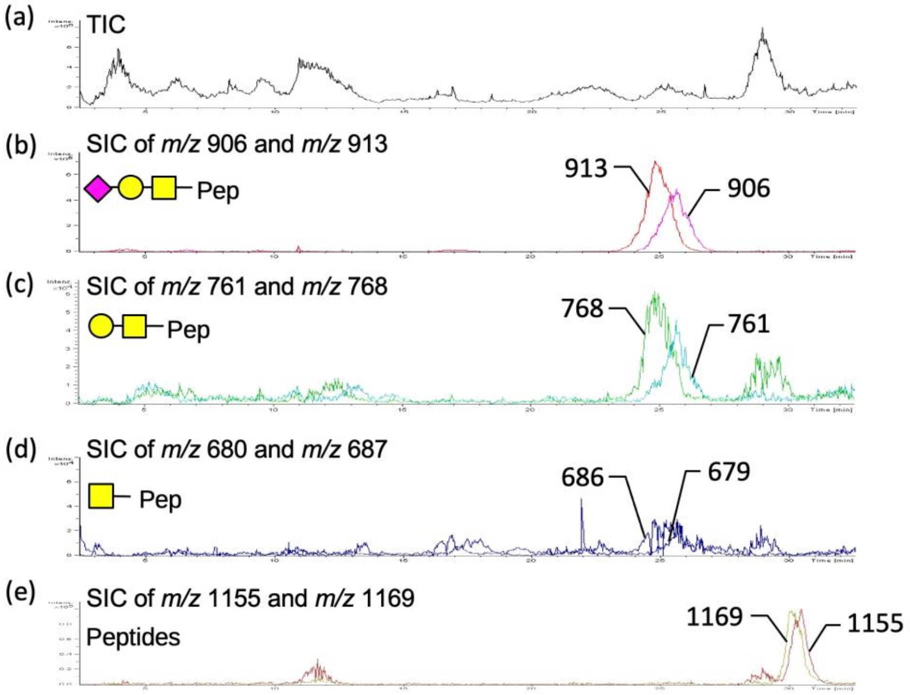

Figure. S2. Total ion chromatogram (TIC) and single ion chromatograms (SIC) for glycopeptides and peptide of Gc protein after tryptic digestion.

(a) Total ion chromatogram in positive ion mode. (b) SIC for trisaccharide-carrying peptides (*m*/*z* 906±0.5 and 913±0.5). (c) SIC for disaccharide-carrying peptides (*m*/*z* 761±0.5 and 768±0.5). (d) SIC for monosaccharide-carrying peptides (*m*/*z* 679±0.5 and 686±0.5). (e) SIC for corresponding peptides (*m*/*z* 1155±0.5 and 1169±0.5).

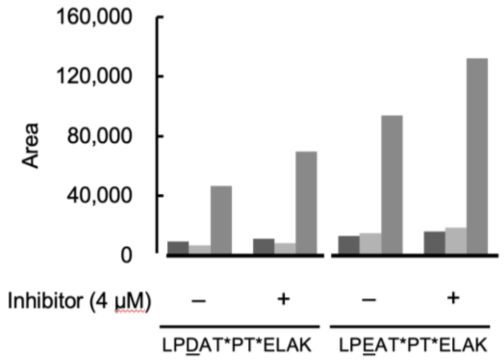

Figure. S3. Effects of a glycosidase inhibitor.

Composition and areas of peptides and glycopeptides were evaluated with and without glycosidase (α-Sia-ase and HepG2 cell extract) treatment followed by tryptic digestion.

Gc Protein solution was first treated with α-Sia-ase for 1 h followed by HepG2 cellular extract in 5 mM $CaCl_2$ containing 50 mM citrate buffer at pH 5.0 with and without inhibitor (4 μM), and incubated at 37 °C for 2 h. The reaction solution was then dialyzed in 50 mM ammonium bicarbonate (1 h × 3 times) for following tryptic digestion.

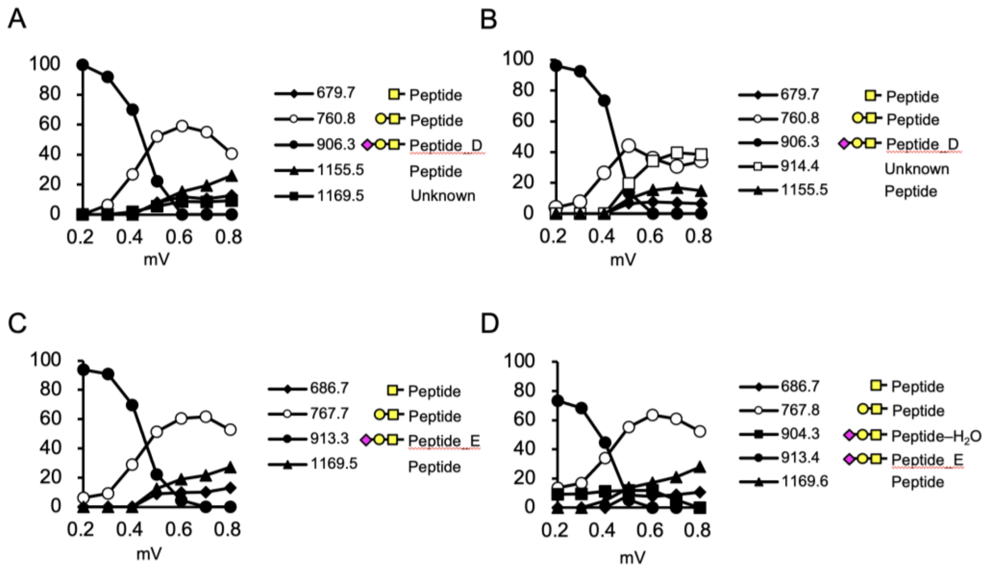

Figure. S4. ERMS of selected ions of glycopeptides.
(a) Precursor ion: *m*/*z* 906.3 in the peak around 13.9 min (Fig. 5a) with its peptide sequence of LPDATPTELAK. (b) Precursor ion: *m*/*z* 906.3 in the peak around 14.8 min (Fig. 5a) with its peptide sequence of LPDATPTELAK. (c) Precursor ion: *m*/*z* 913.3 in the peak around 13.5 min (Fig. 5a) with its peptide sequence of LPEATPTELAK. (d) Precursor ion: *m*/*z* 913.3 in the peak around 14.6 min (Fig. 5a) with its peptide sequence of LPEATPTELAK. Arrangement of the figure is the same as in Fig. 5b.
